# Broadband Terahertz Holography Using Nonlinear Plasmonic Metasurfaces

**DOI:** 10.1021/acs.nanolett.5c05187

**Published:** 2025-12-19

**Authors:** Zixian Hu, Symeon Sideris, Cormac McDonnell, Tal Ellenbogen, Guixin Li

**Affiliations:** ⊥ Department of Materials Science and Engineering, 255310 Southern University of Science and Technology, Shenzhen 518055, China; ‡ Department of Physical Electronics, School of Electrical and Computer Engineering, 26745 Tel-Aviv University, Tel-Aviv 6997801, Israel; § Center for Light-Matter Interaction, Tel-Aviv University, Tel-Aviv 6779801, Israel; ∥ Institute for Applied Optics and Precision Engineering, Southern University of Science and Technology, Shenzhen 518055, China

**Keywords:** Pancharatnam−Berry phase, nonlinear metasurfaces, terahertz technology, multifunctional holography

## Abstract

Terahertz (THz) waves have been successfully utilized in a wide variety of practical applications, such as communications, sensing, and biomedical engineering. Broadband THz emitters with sufficient functionality are of great importance to fully realize the impact of these applications. This demand has stimulated the exploration of THz emitters with the capability of complex field manipulation. Here we report on the development of Pancharatnam–Berry phase nonlinear metasurface emitters for broadband and multifunctional THz holography. Two types of metasurface holograms are designed by which scalar holographic imaging and dual-polarization holographic imaging are experimentally demonstrated in a broadband frequency range up to over 2.0 THz. We show that both broadband THz beam generation and multifunctional THz field manipulation can be realized simultaneously. These demonstrations exhibit the significant potential of nonlinear metasurface emitters as efficient THz sources, which may have important applications in THz frequency information processing.

## Graphical abstract

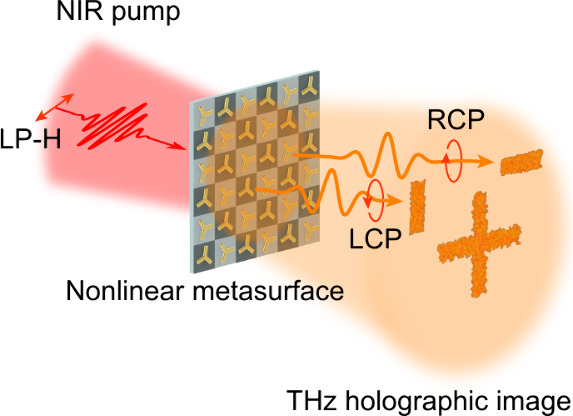

Located between the microwave and infrared spectral regimes, terahertz (THz) waves (0.1–10 THz, 1 THz = 300 μm) have attracted great attention due to their unique properties. THz waves are capable of penetrating through many materials such as paper and plastic, while their relatively low photon energies have fewer ionization effects on biological samples. Since the vibrational and rotational transitions of many materials are located in the THz frequency band, THz waves have great application to the field of spectroscopy.^1,2^ As such, THz waves have been utilized in a wide variety of practical applications such as noninvasive sensing,^3–7^ stand-off inspection and detection,^8,9^ and wireless communications.^10–12^ Therefore, explorations of THz waves are important for both fundamental science and industrial applications, inspiring the development of novel THz devices with new functionalities. In the area of THz technology, generation and manipulation are two important aspects. In particular, an important goal in this area is to achieve a broadband functionality across a wide THz frequency range. The generation of THz waves has been realized by using different strategies, such as nonlinear crystals based on the optical rectification,^13^ photoconductive antennas,^14^ free electron lasers,^15^ quantum cascade lasers,^16^ and spintronic emitters.^17^ On the other hand, manipulation of the THz fields is mainly implemented by using additional optical elements, including polymer diffractive optical elements,^18^ silicon gratings,^19^ kirigami modulators,^20^ and metallic gratings.^21^ To expand and optimize the applications of THz waves, advanced manipulation methods of THz waves with multiple functionalities need to be explored. However, the inherent absorption of materials in additional optical elements is often a major limitation for practical applications. This is especially obvious for the higher-frequency range where typical optical elements consisting of silicon and polymers reflect or absorb most of the field intensity.^22^ Therefore, it is important to develop functional THz emitters with the capability of simultaneously realizing broadband generation and functional manipulation of THz waves. To address this issue, nonlinear plasmonic metasurfaces represent a promising avenue.

Optical metasurfaces are quasi-2D devices that consist of a single layer of spatially varying meta-atoms with subwavelength features. By judiciously designing the geometrical parameters of the meta-atoms, the properties of light passing through them can be well controlled, including amplitude, phase, and polarization.^23–25^ By assembling the meta-atoms in specific arrangements, many on-demand optical functions can be achieved. Thus, optical metasurfaces are well-known for their powerful capability of optical field manipulation.^26^ With their rapid development in the past decade, optical metasurfaces have been widely investigated and have found important applications in the linear optical regime, such as holography, beam steering, and beam focusing.^27–34^ Moreover, due to the strong field localization effects occurring on the plasmonic meta-atoms, an optical metasurface is also an ideal platform for the realization of applications in the nonlinear optical regime, such as frequency conversion,^35–37^ nonlinear wavefront engineering,^38–41^ and nonlinear optical holography.^42–44^ As for the THz regime, recent advances have shown that nonlinear plasmonic metasurfaces are also promising alternatives for the generation of THz waves, with conversion efficiencies approaching 10^–6^, which is explained as a second-order optical rectification process.^45^ A method of binary phase control of THz waves has been demonstrated by flipping the orientation angle of meta-atoms in plasmonic metasurfaces,^46^ which is applied to realize THz Fresnel zone plates.^47^ Full control over the polarization and phase of the emitted THz waves can be achieved through controlling the rotation angles of meta-atoms with 3-fold rotational symmetry (*C*_3_). Demonstrations of THz waves with time-dependent polarizations and amplitude manipulation were realized.^48–51^ In addition, a very recent work reported an optoelectronic metasurface composed of gold nanoantennas fabricated on a graphene layer,^52^ enabling the generation of vectorial THz beams. As an important but challenging application, optical holography, especially holography with high image quality or complex functionality, is ideal to demonstrate the potential of metasurface devices for optical field manipulation. Functional holograms based on optical metasurfaces have been realized in both linear and nonlinear optical regimes,^28,42–44^ which exhibit high efficiency, high image quality, and the capability of multidimensional manipulations. In the THz frequency band, holograms have been realized using methods such as CMOS chip,^53^ continuous wave THz laser systems,^54,55^ all-dielectric metasurfaces,^56^ and Janus thermal metasurfaces.^57^ These demonstrations of THz holography are typically limited to either a single THz frequency or a very limited bandwidth, which is often in the lower region of the THz spectrum. Nonlinear metasurface-based THz holograms may provide the technological leap to further develop THz emitters with more capabilities for optical field manipulation covering the full THz spectrum.

Here, we propose and experimentally demonstrate a method for functional THz holography using nonlinear plasmonic metasurface emitters, realizing scalar holography and dual-polarization holography in the THz region. The general schematic of this scheme is illustrated in Figure 1. The proposed emitters are composed of gold plasmonic *C*_3_ meta-atoms, through which THz waves can be generated under the illumination of a femtosecond (fs) laser with both left and right circular polarization (LCP/RCP) components.^48^ According to the concept of the Pancharatnam–Berry (P–B) phase, the phase of the emitted THz waves can be fully controlled as a result of the high-precision manipulation of the meta-atoms at the extremely subwavelength scale. With these foundations, we design phase-only type metasurface holograms by using a modified Gerchberg–Saxton (G–S) algorithm for realizing two-dimensional scalar THz holography. In addition, by arranging the meta-atoms in an interleaving scheme, we encode two independent images into a single metasurface hologram, which have different circular polarization states and are separately displayed in the spatial domain. It is experimentally demonstrated that both the intensity and polarization distributions of the generated THz holographic images are well-controlled, while a wide working frequency band is observed ranging from 0.6 to 2.0 THz. The proposed metasurface-based emitter for functional THz holography represents a novel avenue for the multidimensional manipulation of the THz fields, exhibiting advantages in high precision and flexibility in encoding. In principle, this method can be developed to achieve more complicated functions, such as vectorial holography and multichannel polarization multiplexing, which may find important applications in ultracompact and multifunctional THz sources and promote the development of THz technologies.

**Figure 1:**
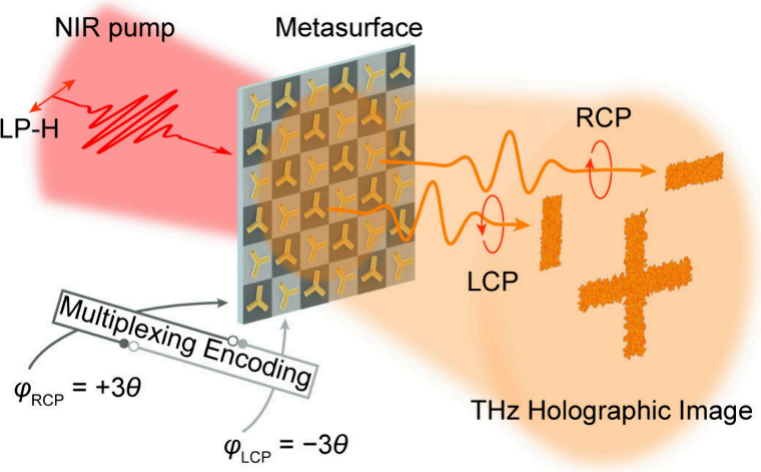
Schematic illustration of broadband THz holography using nonlinear plasmonic metasurfaces. Using an interleaving encoding method, the plasmonic metasurface THz hologram enables the broadband generation of THz waves and multifunctional holography. NIR, near-infrared; LP-H, horizontal linear polarization; RCP/LCP, right or left circular polarization, represents the polarization state of the generated THz waves; φ, holographic phase; θ, orientation angle of the meta-atom.

## THz Generation and P–B Phase Control on *C*_3_ Meta-atoms

The THz emitters are plasmonic metasurfaces that consist of gold *C*_3_ meta-atoms fabricated on a 15 nm-thick indium-tin-oxide (ITO) coated glass substrate. The *C*_3_ meta-atoms, whose arm length and arm width are 195 and 70 nm, respectively, are arranged in a square lattice with a period of 500 nm, as shown in Figure 2a. Here, the *C*_3_ gold plasmonic meta-atom has inversion symmetry breaking property; therefore, the THz wave generation process is allowed.^48^ For a *C*_3_ meta-atom with an orientation angle θ, the nonlinear dipole moment during the second-order optical rectification can be described by the following equation: *p*_–σ_ = α_0_ *ε*_0_ *E*_*σ*_(ω_1_)*E*_–σ_^*^(ω_2_)*e* ^3*iσθ*^, where α_0_ is the nonlinear polarizability of the meta-atom; *ε*_0_ is the vacuum permittivity; σ = ±1 represents the LCP or RCP state of the FW; ω_1_ and ω_2_ are the angular frequencies of the two interacting waves, where ω_THz_ = ω_1_ – ω_2_; and 3*σθ* represents the nonlinear P–B phase. Therefore, the generated THz waves may contain RCP and LCP components, which carry nonlinear P–B phases of +3θ and – 3θ, respectively, as illustrated in Figure 2b. Broadband THz radiation can be generated from the *C*_3_ plasmonic metasurfaces under the pumping of near-infrared (NIR) ultrashort laser pulses, which has been verified in previous works.^48^ The NIR spectral region is chosen as the pumping wavelength in order to illuminate the sample at the epsilon-near-zero (ENZ) region of ITO, which results in enhanced field confinement and subsequent increased THz emission.^58^ In previous studies, it is shown that the ENZ effect in the ITO film significantly enhances the second-order nonlinearity.^59^ Recent advances have shown that nonlinear plasmonic metasurface coupled to an ITO ENZ thin film allows the generation of THz waves with conversion efficiency approaching 10^–6^, which is explained mainly as a second-order optical rectification process.^45^ This is in contrast to plasmonic metasurfaces on glass substrate alone, which primarily emit THz due to the pondermotive acceleration of photoejected electrons.^58^ The conversion efficiency in this work is lower than that achieved with other high-power THz sources. However, as a trade-off, these sources are often extremely limited in emission bandwidth, with subsequent optical elements for wave manipulation operating in only a small wavelength range. In addition, metasurface THz emitters have their advantages in miniaturization and integration. The conversion efficiency of the plasmonic metasurface THz emitters can be further improved if combined with other material platforms, such as lithium niobate.^60^ It is notable that the fundamental waves (FWs) must contain both LCP and RCP components, otherwise this nonlinear optical process is forbidden.^48^ In the experiments, the plasmonic metasurfaces are pumped using horizontally polarized FWs (Figure 1). According to the results of our previous work,^50^ the polarization direction of the FW can be used to control the polarization direction of the generated THz wave, but it has negligible influence on the efficiency of THz emission. As for the following design of the metasurface holograms, the phase distributions introduced into the RCP and LCP THz components are determined only by the orientation angles of the *C*_3_ meta-atoms. Thus, the emission pattern in the THz holographic image remains unaffected by the polarization direction of the FW. As for the conversion efficiency, here, we can give an estimation on the *C*_3_ plasmonic metasurface emitters in this work. At the pump intensities used in this study (∼10 GW/cm^2^), the measured NIR to THz conversion efficiency of the gold–ITO metasurfaces is about 10^–6^ as shown in previous studies.^45^ Therefore, the energy conversion efficiency of the THz metasurface emitters in this work is estimated to be on the scale of 10^–7^ to 10^–6^, which is influenced by the random holographic phase distribution across the metasurface. This random phase profile induces both constructive and destructive interferences of the THz waves, thereby determining the final conversion efficiency.

**Figure 2:**
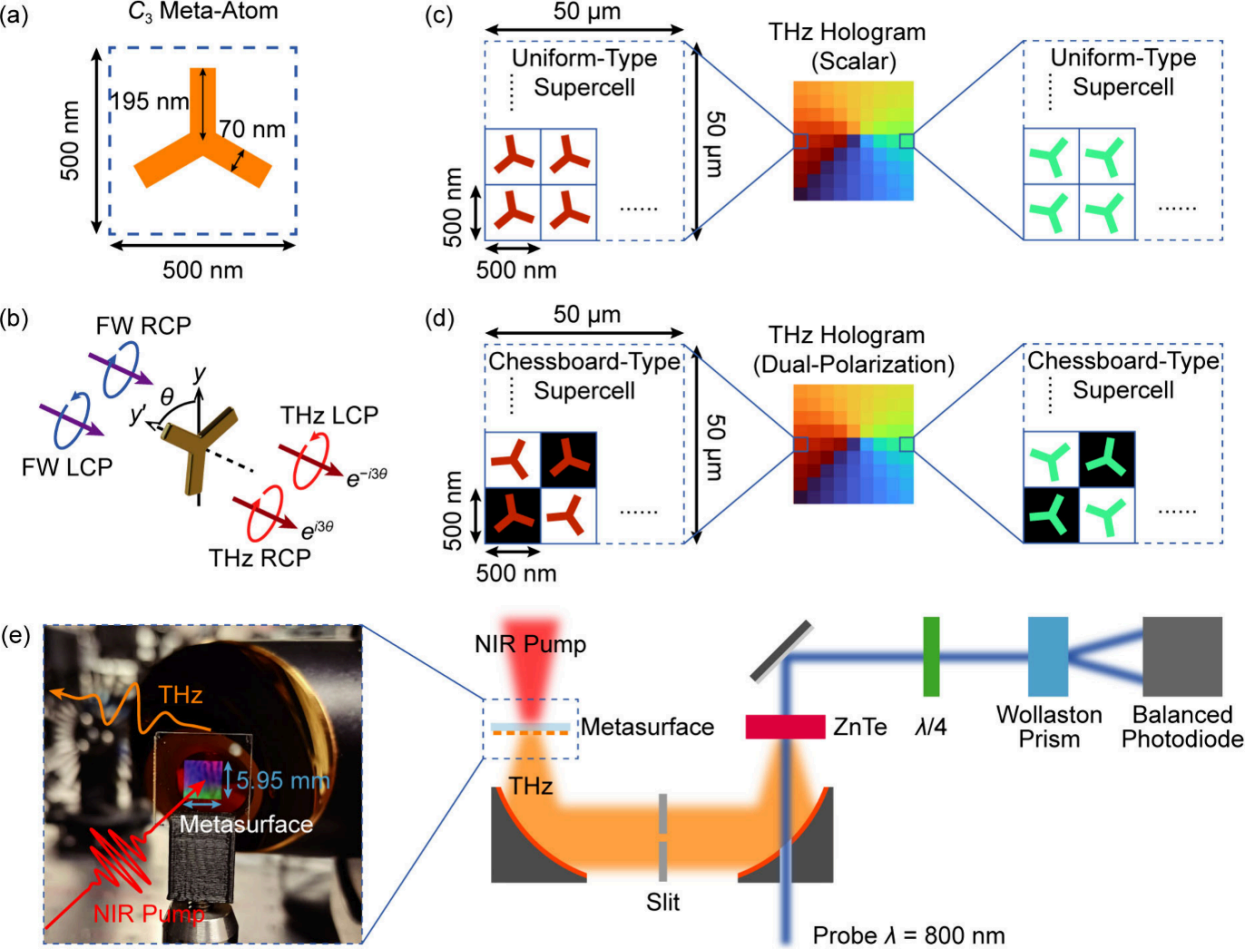
Schematics for the design and characterization of the plasmonic metasurface THz holograms. (a) The meta-atom with 3-fold rotational symmetry (*C*_3_). (b) Schematic diagram of THz generation and Pancharatnam–Berry phase on the gold *C*_3_ meta-atom. θ is the orientation angle of the meta-atom. (c) Schematic illustration of the metasurface hologram for scalar THz holography. The uniform-type supercell is composed of 100 × 100 *C*_3_ meta-atoms with the same orientation angle, serving as a pixel for phase manipulation on the THz hologram. (d) Schematic illustration of the metasurface hologram for dual-polarization THz holography, in which the supercell is constructed in a chessboard-type scheme. Two kinds of *C*_3_ meta-atoms with different orientation angles are arranged in the supercell with an interleaving configuration. They are designed to introduce holographic phase profiles for the RCP and LCP components in THz waves, which are marked by the black and white grids in the schematic diagram. (e) Schematic illustration of the experimental setup of the time-domain THz spectroscopy. The inset photo shows the metasurface sample in the measurement, which has a working area of about 5.95 mm × 5.95 mm. ZnTe, zinc telluride nonlinear crystal; λ/4, quarter-wave plate; RCP/LCP, right or left circular polarization.

## Design of the Metasurface THz Hologram

To obtain the required phase profile for generating the desired holographic images, we employ a modified G–S algorithm based on Fresnel diffraction.^43,61,62^ Accounting for the phase accumulation due to the propagation over a distance *z*, this modified version of the G–S algorithm ensures the convergence to a phase profile with smooth transitions which correspond to propagating modes (see section S1 and Figure S1 in the Supporting Information for details and the logic diagram). The calculated phase profiles are introduced by using *C*_3_ meta-atoms based on the concept of P–B phase. The appropriate size of the supercell for phase manipulation is much larger than the period of the meta-atoms to ensure well-shaped radiation in the far field. In this case, an array composed of 100 × 100 *C*_3_ meta-atoms with the same orientation angle is chosen as the fundamental supercell for phase manipulation, which is named as the uniform-type supercell (Figure 2c). Therefore, the overall size of the supercell is 50 μm × 50 μm, and the area size of the THz metasurface is 5.95 mm × 5.95 mm, containing 119 × 119 supercells. It should be noted that the selection of the size of supercells is estimated and determined based on the simulations, considering the balance between the pattern shape and intensity uniformity of the generated holographic image (see section S2 and Figure S2 in the Supporting Information for details). The first method introduced above is used to realize scalar THz holography, which can generate a centrosymmetric holographic image consisting of a defined intensity profile across the generated frequencies. However, it has a random linear polarization distribution that is hard to control. To realize full control of the THz fields, the property of the P–B phase metasurface is then utilized to introduce phase distributions with the same value but opposite signs for the RCP and LCP components, enabling the realization of dual-polarization THz holography. As shown in Figure 2d, the meta-atoms in the supercell are arranged in a chessboard-type configuration. For each supercell, the area size is still 50 μm × 50 μm, and the meta-atoms in the black and white grids have different orientation angles, introducing independent phase distributions for the RCP and LCP components of the generated THz waves. Hence, by using this method, two spatially separated polarization channels are obtained in which two different THz holographic images can be simultaneously encoded. Finally, the performances of these two types of metasurface THz holograms are characterized using a time-domain THz spectroscopy setup, which is illustrated in Figure 2e.

## Scalar THz Holography

To verify the ability to realize scalar THz holography, a metasurface hologram with uniform-type supercells is designed and fabricated. The target image is shown in Figure 3a, which consists of a centrosymmetric cross pattern. The required phase profile is shown in Figure 3b, which is calculated by using the aforementioned holography algorithm. Figure 3c shows the calculated electric field magnitude distribution of the holographic image after a propagation of 50.8 mm to the collimation plane. The working frequency in the calculation is set to be 1.0 THz. Through a standard nanofabrication process (see Methods for details), the plasmonic metasurface is prepared, and its scanning electron microscope (SEM) image is shown in Figure 3d. Figure 3e shows the measured NIR transmission spectra of the metasurface, which exhibits a dip in the transmission spectrum over the wavelength range from 1100 to 1600 nm, indicating a resonant response in the ENZ region of the ITO film. Therefore, the central wavelength of the fs laser to excite the emission of THz waves from the metasurface is set to be 1300 nm. The spectrum of the emitted THz waves in the experiment is shown in Figure 3f, exhibiting a broadband frequency range from 0.6 to over 2.0 THz. THz waves at higher frequencies up to the ZnTe crystal detection limit of 2.5 THz are also generated (Figure S3 in the Supporting Information). Frequencies above 2.5 THz are expected to be emitted but are not detected in the ZnTe crystal due to phase mismatch between the probe pulse and the THz pulse. The spatio-spectral properties of the generated THz holographic images are examined using time-domain spectroscopy (see Methods for a full experimental description). Figure 3g–j shows the electric field magnitude distributions of the generated THz holographic images in the collimated plane, corresponding to the selected frequency components of 1.0, 1.2, 1.4, and 1.6 THz, respectively. The measured THz holographic images show a well-defined cross shape over the whole frequency range of the generated THz waves, which agrees with our design. Meanwhile, it can be observed that the size of the holographic images decreases as the frequency increases, since the components with higher frequencies in THz waves have smaller diffraction angles. This phenomenon is also verified by our calculations (Figure S4 in the Supporting Information).

**Figure 3:**
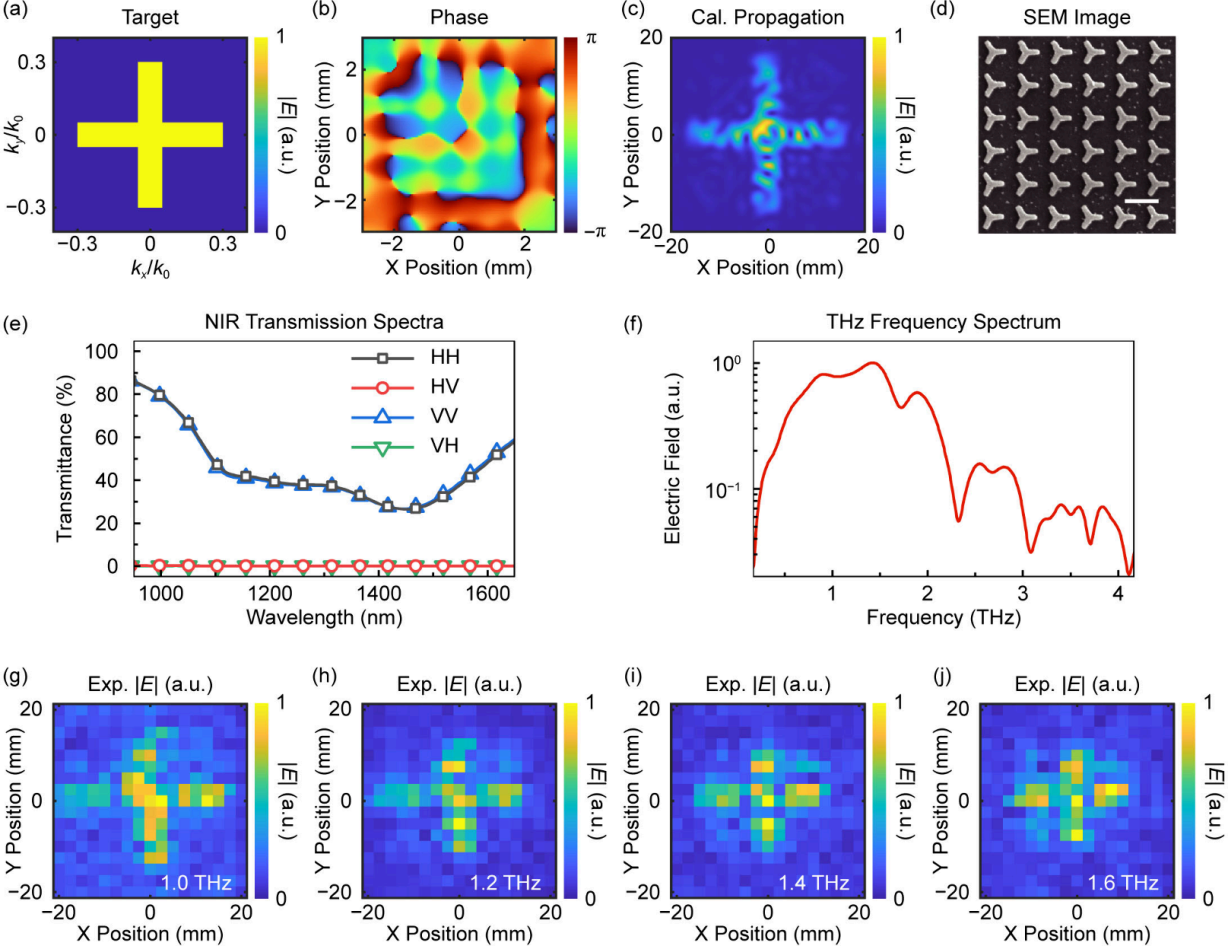
Scalar THz holography by using the metasurface emitter with uniform-type supercells. (a and b) The target image (a) and the required phase distribution (b) of the metasurface for generating the holographic image. (c) The calculated THz holographic image after propagation (distance 50.8 mm). The working frequency in the design and calculation is set to be 1.0 THz. (d) The scanning electron microscopy image of the plasmonic metasurface for scalar holography (Scale bar, 500 nm). (e) The measured polarization-resolved near-infrared (NIR) transmission spectra of the plasmonic metasurface for scalar holography. H, horizontal polarization; V, vertical polarization. “HH”, “HV”, “VV”, and “VH” are the four kinds of polarization combinations in the measurement, in which the first and second characters denote the polarization states of the incident and transmitted light, respectively. (f) The frequency spectrum of the generated THz waves, ranging from 0.6 THz to over 4.0 THz. The sampling position is at the center of the holographic image. (g–j) The measured electric fields of the THz holographic images at frequencies of 1.0, 1.2, 1.4, and 1.6 THz. Cal, calculated results; Exp, experimental results.

## Dual-Polarization THz Holography

Next, the ability to realize THz holography with two polarization channels is investigated, which is based on a metasurface hologram with chessboard-type supercells. Figure 4a,d shows the target electric field magnitude distribution of the RCP and LCP components in the THz holographic image, whose shapes are horizontal and vertical bars (“H-bar” and “V-bar”), respectively. By using the same numerical method as previously introduced, the required phase profiles and the calculated THz holographic images are obtained, which are shown in Figure 4b,e and Figure 4c,f, respectively. The plasmonic metasurface for dual-polarization holography is then fabricated, and the SEM image and measured NIR transmission spectra are shown in Figure 4g,h. Under the pumping of a linearly polarized NIR fs laser (central wavelength 1300 nm), the holographic images in the generated THz waves are measured. The electric field magnitude distributions of the *E*_*x*_ and *E*_*y*_ components are shown in Figure 5a–h, at the selected frequencies of 1.0, 1.2, 1.6, and 2.0 THz (results at other frequencies are shown in Figure S5 of the Supporting Information). It should be noted that the patterns of the horizontal bar at the top right and the vertical bar at the top left are the target holographic images, which are marked by the red dotted boxes and orange dashed boxes, respectively. The twin images at the bottom left and bottom right are not included in the design but come from the opposite nonlinear P–B phase. This phenomenon can be observed in most of the metasurface optical holography based on the P–B phase.^43^ In general, the quality of the measured THz images shows some distortion relative to the ideal electric field distribution. This is due to the challenges of measuring such a large-area THz hologram using a pixel-by-pixel aperture scanning method. The aperture in conjunction with the large area of the THz electric field distribution leads to some constraints on the measured signal-to-noise ratio as well as potentially adding diffraction effects on the transmitted field through the aperture. Another reason is that the pixel number of the hologram is only 119 × 119, which is insufficient for high-quality holography. Therefore, from the perspective of the metasurface design, one possible solution is to enlarge the size of the metasurface to provide more manipulation pixels in the hologram. In addition, the decrease of the holographic image size with the increase of frequency is observed, which is similar to that in the scalar design. It is verified by our calculations (Figures S7 and S8 in the Supporting Information). This phenomenon is also verified by the measured THz spectra, which are shown in Figure S9 of the Supporting Information. As the sampling position moves from the edge to the center of the holographic image, the central frequency of the extracted spectra gradually increases. To determine the circular polarization states of the THz holographic images measured in the experiment, we examined the time-domain electric fields of the *E*_*x*_ and *E*_*y*_ components. Figure 5i,j shows the *E*_*x*_ and *E*_*y*_ spatiotemporal fields across the *x*-dimension at a fixed scanning position on the *y*-axis, which is marked by the white dashed lines in Figure 5a–h. The THz waves are diffracted to both the positive and negative directions along the *x*-axis, corresponding to the RCP and LCP components. Figure 5k,l shows the cross sections of *E*_*x*_ and *E*_*y*_ spatiotemporal fields at the fixed *x* spatial positions taken from Figure 5i,j, which are indicated by the red dotted lines and orange dashed lines, respectively. In the case of a THz wave in the positive diffraction direction (Figure 5k), a negative π/2 phase shift is observed between the *E*_*x*_ and *E*_*y*_ components of the electric field, indicating the RCP polarization state. In contrast, for the THz wave in the negative diffraction direction (Figure 5l), a positive π/2 phase shift is observed, indicating the LCP polarization state. It can be found that the polarization states of the THz holographic images agree well with those in the design.

**Figure 4:**
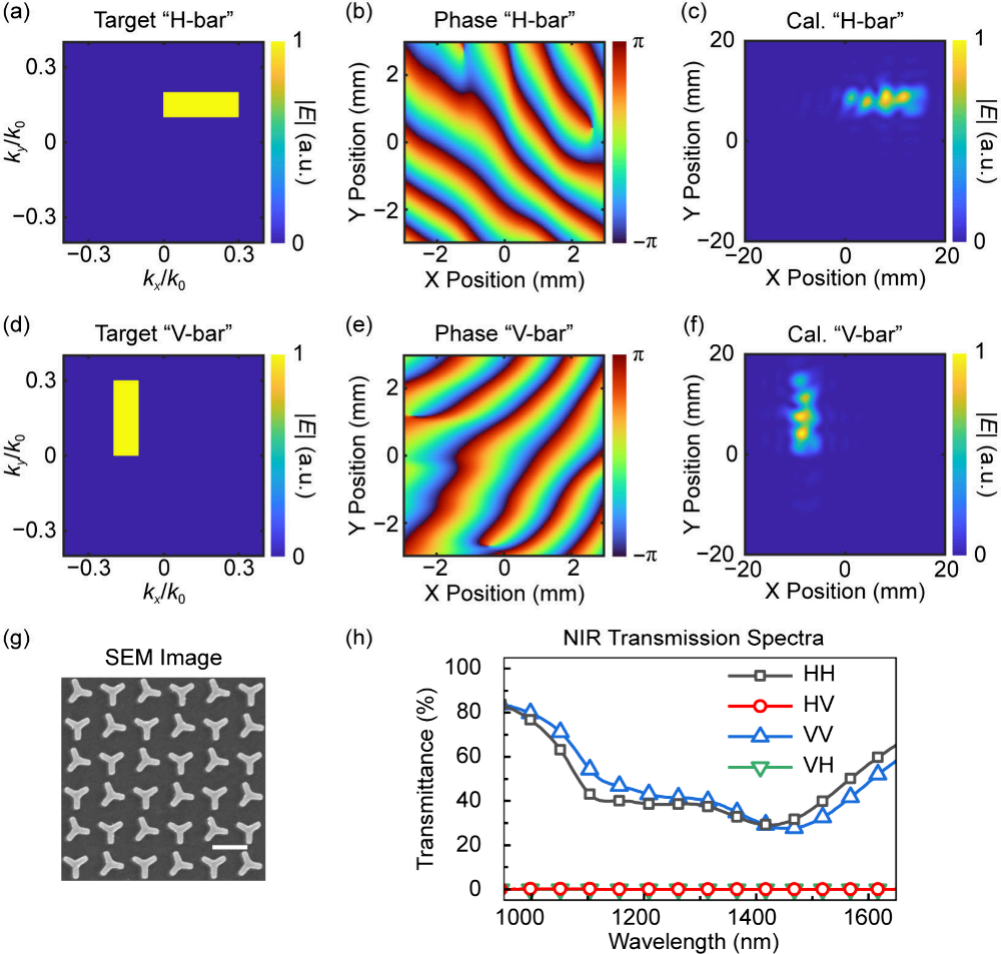
Design and basic characterizations of the metasurface emitter for dual-polarization THz holography. (a and d) The target images for THz waves with RCP (a) and LCP (d) states. (b and e) The required phase distributions of the metasurface for generating the THz holographic images with RCP (b) and LCP (e) states. (c and f) The calculated THz holographic images after propagation (distance 50.8 mm) for THz waves with RCP (c) and LCP (f) states. The working frequency in the design and calculation is set to be 1.0 THz. (g) The scanning electron microscopy image of the plasmonic metasurface for dual-polarization holography (Scale bar, 500 nm). (h) The measured polarization-resolved near-infrared (NIR) transmission spectra of the plasmonic metasurface for dual-polarization holography. H, horizontal polarization; V, vertical polarization. “HH”, “HV”, “VV”, and “VH” are the four kinds of polarization combinations in the measurement, in which the first and second characters denote the polarization states of the incident and transmitted light, respectively. RCP/LCP, right or left circular polarization.

**Figure 5:**
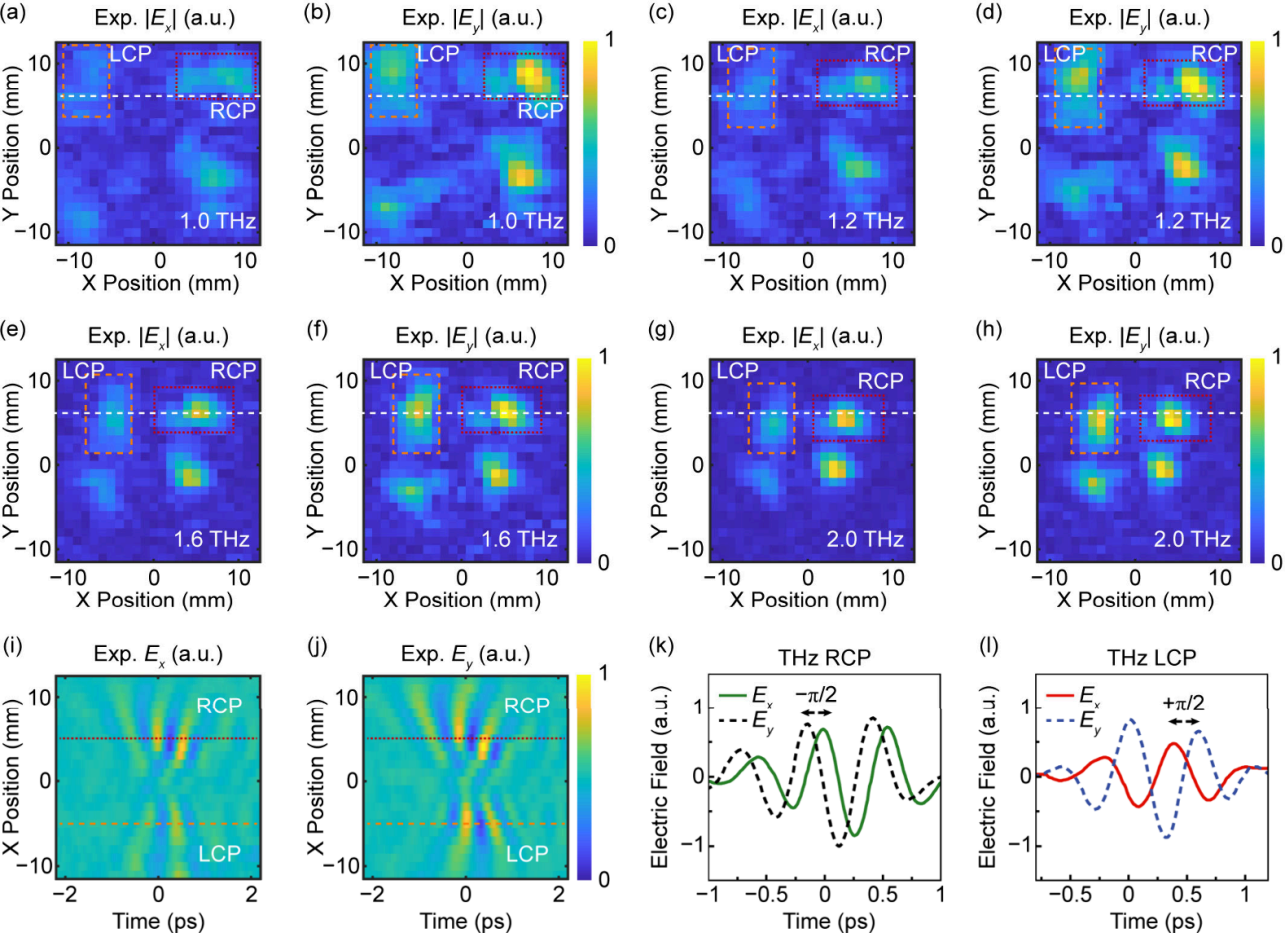
Dual-polarization THz holography using the metasurface emitter. (a, c, e and g) The measured *E*_*x*_ electric fields of the THz holographic image at frequencies of 1.0, 1.2, 1.6, and 2.0 THz, respectively. (b, d, f and h) The measured *E*_*y*_ electric fields of the THz holographic image at frequencies of 1.0, 1.2, 1.6, and 2.0 THz, respectively. The target images with shapes of the horizontal and vertical bars, corresponding to the RCP and LCP components in the THz waves, are indicated by using the red dotted boxes and orange dashed boxes, respectively. (i and j) The *E*_*x*_ and *E*_*y*_ components of the THz temporal fields, exhibiting multicycle pulses diffracted to the positive (RCP component) and negative (LCP component) directions along the *x*-axis. Here the scanning position on the *y*-axis is fixed, which is indicated by the white dashed lines in panels a–h. (k and l) The relative phase shift between the *E*_*x*_ and *E*_*y*_ electric fields of the THz waves in the positive (k) and negative (l) diffraction directions. For the THz wave in the positive diffraction direction, a negative phase shift from *E*_*x*_ to *E*_*y*_ is observed, indicating the RCP state. In contrast, a positive phase shift is observed for the THz wave in the negative diffraction direction, indicating the LCP state. The selected scanning positions on the *x*-axis in the positive and negative diffraction directions are indicated in panels i and j by using red dotted lines and orange dashed lines, respectively. Cal, calculated results; Exp, experimental results; RCP/LCP, right or left circular polarization.

In conclusion, we demonstrate the application of nonlinear P–B phase plasmonic metasurfaces to design holographic broadband THz emitters. By controlling the in-plane orientation angle of the *C*_3_ meta-atoms, we can precisely introduce the holographic phase distributions. Based on a modified G–S algorithm, the phase-type THz holograms are calculated with a pixel size of 50 μm × 50 μm. The phase manipulation pixels are replaced by supercells that consist of meta-atoms with the same orientation angle. With this strategy, a metasurface THz emitter for scalar holography is prepared and characterized. Under the pumping of NIR fs laser pulses with linear polarization, the holographic image with a centrosymmetric cross pattern is generated, which is well-shaped in a broad frequency range from 0.6 up to 2.0 THz. In addition, by using the interleaving method to construct the supercells, two independent THz holographic images can be encoded into two spatially separated polarization channels. Based on this concept, we design and fabricate a metasurface THz emitter for realizing dual-polarization THz holography, in which the holographic image contains patterns with opposite circular polarization states. These approaches provide solutions to circumvent the challenge of achieving generation and effective manipulation of THz waves with a single device. The powerful capability of the metasurface THz emitters for nonlinear field manipulation makes them an ideal platform for designing in situ functional THz devices. Moreover, metasurfaces are remarkable for their high flexibility in design, which means that the functionality of the metasurface THz emitters can be further extended. The use of nonlinear metasurfaces also overcomes the typically narrow bandwidth associated with previous methods of realizing THz holograms. In this case, the bandwidth is limited by the pump pulse duration, with the metasurfaces holding the potential for generating frequencies above 5.0 THz and beyond. Therefore, many metasurface-based holographic applications with complex functions can be extended to the THz regime, such as vectorial holography, polarization switching, and grayscale imaging.

## Methods

### Fabrication of Plasmonic Metasurfaces

The plasmonic metasurface is fabricated through a standard bottom-up electron beam lithography method. First, a commercially available 0.7 mm-thick glass substrate coated with 15 nm-thick ITO film is divided into pieces with a desired size (15 mm × 15 mm) by using a dicing machine. The substrate is immersed in acetone solution, isopropanol solution, and deionized water, respectively, and cleaned using ultrasonication. The cleaned substrate is then dried using the nitrogen stream and baked at 180 °C for 3 min. Then a layer of positive electron resist (ZEP520A) is spin-coated on the prepared substrate and baked at 180 °C for 3 min. The designed metasurface patterns are transferred to the resist layer through electron beam lithography followed by the developing process. Afterward, a 30 nm thick gold film is deposited on the sample using an electron beam evaporator. Finally, the gold plasmonic metasurface on the ITO-coated glass is obtained after a lift-off process.

### Linear Optical Properties of the Plasmonic Metasurfaces

The linear optical response of the plasmonic metasurfaces is characterized by using a homemade linear transmission experimental system. A continuous-wave halogen lamp (Alltion XD-301-150W) is utilized as the light source, and an NIR spectrometer (Ocean Optics Flame-NIR) is used to measure the transmission spectra. The NIR transmission spectra of the plasmonic metasurfaces are obtained under four combinations of linear polarization states of the incident and transmitted light. The linearly polarized light is focused using an objective lens (N.A. = 0.25) and normally incident onto the metasurface from the substrate side. The transmitted light is collected by using another objective lens with the same N.A., which is analyzed by using a linear polarizer. The transmitted light is then focused by using a convex lens (*f* = 100 mm) and coupled to the fiber of the spectrometer for the characterization of spectra.

### Time-Domain THz Spectroscopy

The generation and detection of the THz holographic images are performed using a time-domain THz spectroscopy setup, which allows the extraction of the spatiotemporal and spatio-spectral information on the holographic images. The general setup is based on a pump–probe scheme. The laser (Spectra Physics Solstice Ace) emitted 800 nm pulses with a pulse duration of 35 fs, which were split into the pump and probe lines. On the pump line, the 800 nm pulses are used as the input of an optical parametric amplifier (OPA) laser system, emitting pulses in the NIR region with a pulse duration of approximately 50 fs. A wavelength of 1300 nm is used for all experiments. After the OPA output, a number of optical elements are used to control the laser polarization, power, and beam size. Beam expanding optics are used to increase the 3 mm beam output to a 1/e^2^ diameter of 6 mm. A pump power of 80 mW is used, giving a peak power density of ∼10 GW/cm^2^. The metasurface is placed at the focal point of an off-axis parabolic mirror (*d*_oam_ = 50 mm, *f* = 50 mm), which collimates the generated THz holographic images. A Teflon filter is used to remove the pump pulse from the collection area. After collimation, a square aperture (*d*_ap_ = 2 mm) is raster scanned in the *x*-*y* plane and for both *x* and *y* THz polarizations to extract the polarization-dependent spatial information on the holographic images. For the scalar hologram, 17 × 17 pixels are used, giving a total image size of 40 mm × 40 mm. For the dual-polarization hologram, 24 × 24 pixels are scanned with a higher resolution, with a total image size of 23 mm × 23 mm. This higher resolution is used to better sample the features of the dual polarization hologram. After transmission through the aperture, the THz beam is focused into a zinc telluride (ZnTe) nonlinear crystal (*d*_ZnTe_ = 500 μm) for electro-optic detection using the second off-axis parabolic mirror (*f* = 50 mm). The ZnTe crystal detects frequencies up to a maximum of 2.5 THz. In the probe line, the 800 nm pulses are sent to a motorized delay stage, which controls the time overlap between the probe and THz pulse. After the stage, the probe pulses are lightly focused into the ZnTe nonlinear crystal with the THz pulse. The amplitude and phase of the THz pulses are extracted using electro-optic detection consisting of a Wollaston prism and a balanced photodetector. The signal is averaged over 300 ms by using a lock-in amplifier.

## Supplementary Material



## Notes

Modified Gerchberg–Saxton algorithm for THz holography; effects of pixel size on the THz holography; measured scalar THz holographic images; calculated scalar THz holographic images; measured dual-polarization THz holographic images; circular polarization resolved dual-polarization THz holographic images; calculated dual-polarization THz holographic images; measured spectra of THz waves in dual-polarization holography; SI references (PDF)
