# Supplementary material for: Broadband Terahertz Holography Using Nonlinear Plasmonic Metasurfaces
Source: Nano Lett. 2025 Dec 19;25(52):18108–16. doi: 10.1021/acs.nanolett.5c05187 (PMC12766728; doi:10.1021/acs.nanolett.5c05187)
Supplement: Supplementary file 1 [file nl5c05187_si_001.pdf]

Supporting Information

Broadband Terahertz Holography Using Nonlinear Plasmonic Metasurfaces

Zixian Hu[1,†], *Symeon Sideris*[2,3,†], *Cormac McDonnell*[2,3,†], *Tal Ellenbogen*[2,3,*], and *Guixin Li*[1,4,*]

[1]Department of Materials Science and Engineering, Southern University of Science and Technology, Shenzhen 518055, China

[2]Department of Physical Electronics, School of Electrical and Computer Engineering, Tel-Aviv University, Tel-Aviv 6997801, Israel

[3]Center for Light-Matter Interaction, Tel-Aviv University, Tel-Aviv 6779801, Israel

[4]Institute for Applied Optics and Precision Engineering, Southern University of Science and Technology, Shenzhen 518055, China

[*]Email: tellenbogen@tauex.tau.ac.il (T.E.); ligx@sustech.edu.cn (G.L.)

[†]These authors contributed equally to this work

KEYWORDS: *Pancharatnam–Berry phase*, *nonlinear metasurfaces*, *terahertz technology*, *multifunctional holography*

Contents:

S1: Modified Gerchberg–Saxton Algorithm for THz Holography

S2: Effects of Pixel Size on the THz Holography

S3: Measured Scalar THz Holographic Images

S4: Calculated Scalar THz Holographic Images

S5: Measured Dual-Polarization THz Holographic Images

S6: Circular Polarization Resolved Dual-Polarization THz Holographic Images

S7: Calculated Dual-Polarization THz Holographic Images

S8: Measured Spectra of THz Waves in Dual-Polarization Holography

SI References

S1: Modified Gerchberg–Saxton Algorithm for THz Holography

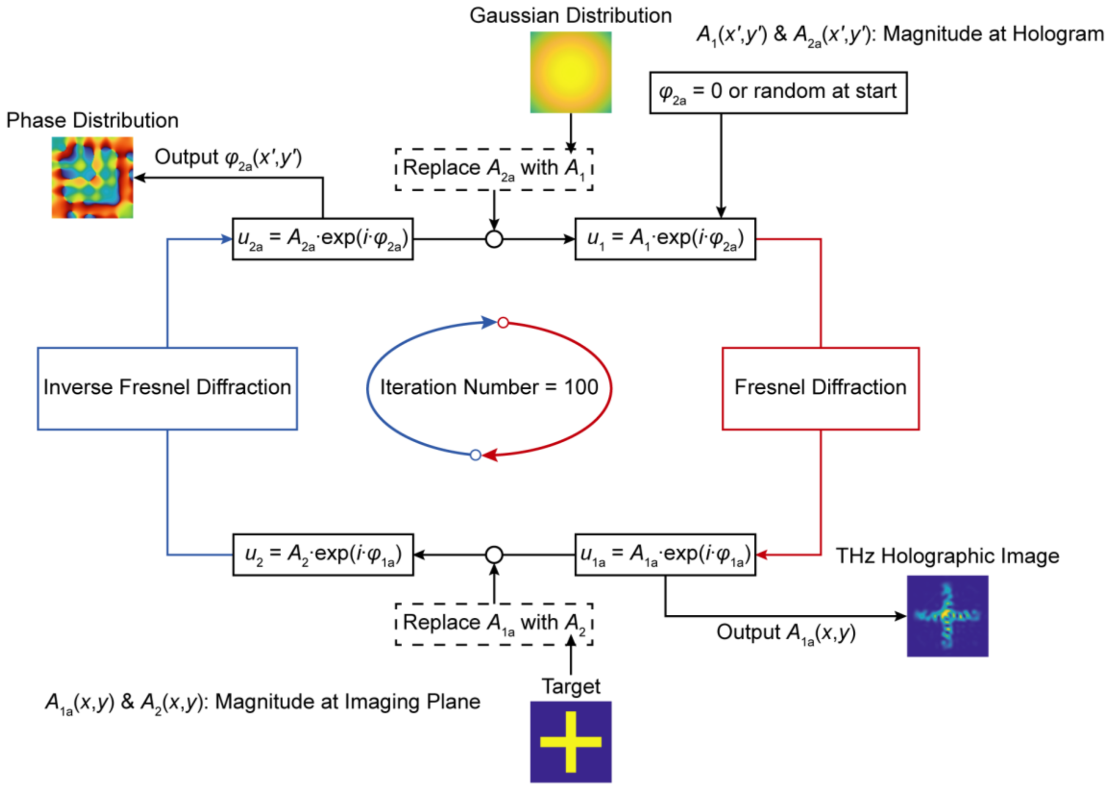

Figure S1. Logic diagram of the modified Gerchberg–Saxton algorithm. Based on the Fresnel diffraction, the phase accumulated during the THz wave propagation is considered in the algorithm. As the propagation depending on the working frequency and the imaging distance is well-defined, this modified G–S algorithm ensures the convergence to a phase profile with smooth transitions. The coordinates of (x',y') and (x,y) represent the electric fields of the THz waves at the hologram and the imaging planes, respectively.

The schematic illustration of the modified Gerchberg–Saxton (G–S) algorithm is shown in Figure S1. The algorithm is mainly based on the Fresnel diffraction integral,[1] which can be represented as:

$$u_{1a}(x,y,z) = \frac{e^{ikz}}{i\lambda z}\int\int_{-\infty}^{+\infty} u_1(x',y',0)\cdot e^{\frac{ik}{2z}\left[(x-x')^2+(y-y')^2\right]}dx'dy' \tag{S1}$$

where λ is the working wavelength of the THz wave, $k = 2\pi/\lambda$ is the wavenumber, and z is the imaging distance. The electric fields at the hologram and the imaging planes are represented by $u_1(x',y',0)$ and, $u_{1a}(x,y,z)$, respectively. The magnitude of the electric field on the hologram has a Gaussian distribution, which is determined by the incident laser beam. It should be noted that

this modified G–S algorithm is performed based on the electric fields in vacuum, where the frequency is set to be 1.0 THz. Therefore, the metasurface in calculation is regarded as a phase-only type hologram. Other parameters in the simulation are as follows: The propagation distance between the hologram plane and imaging plane is set to be 50.8 mm; the pixel number of the hologram is 119 × 119, and the pixel size is selected to be 50 μm × 50 μm after optimization, therefore the size of hologram is 5.95 mm × 5.95 mm; the waist diameter of the incident beam is set to be 4 mm.

S2: Effects of Pixel Size on the THz Holography

In the design of the metasurface THz holograms, to select the supercell with a proper size, four kinds of pixel size are used in the design. The calculated phase profiles for generating the desired cross-pattern holographic image are shown in Figure S2a–d, corresponding to the designs with pixel sizes of 25 μm × 25 μm, 50 μm × 50 μm, 100 μm × 100 μm and 200 μm × 200 μm, respectively. Performances of the designs with different pixel sizes are examined by calculating the holographic images after propagation, which are shown in Figure S2e–h. The working frequency and propagation distance are 1.0 THz and 50.8 mm, respectively. With the decreasing of the pixel size, the shape of the intensity profile in the holographic images becomes smoother and closer to the desired cross-pattern. However, a relatively inhomogeneous intensity distribution especially on the four "arms" is observed in the calculation results with the minimum pixel size of 25 × 25 μm. To balance the shape of the pattern and uniformity of the intensity profile, the pixel size of 50 × 50 μm is selected.

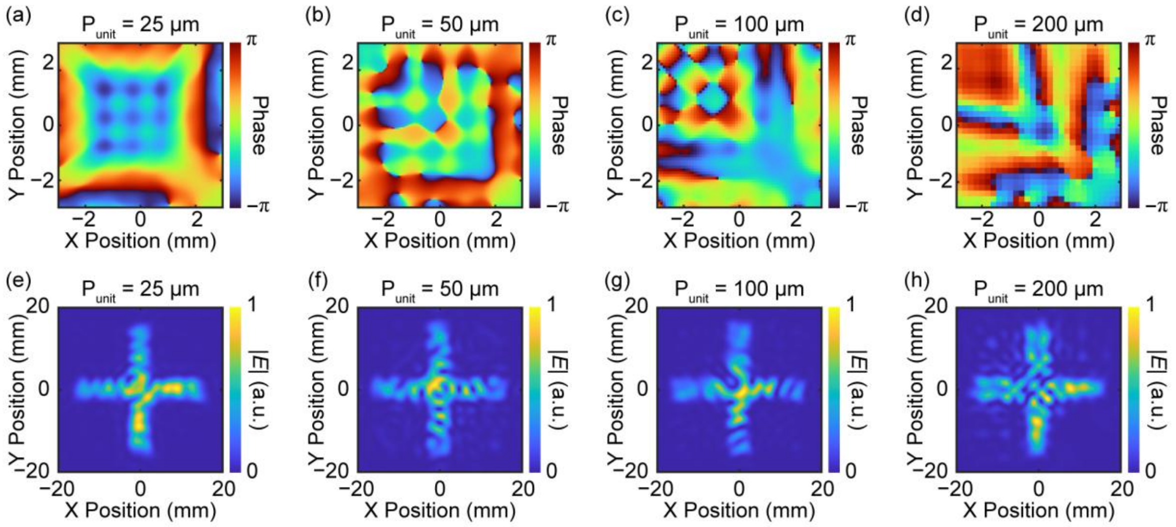

Figure S2. Effects of pixel size in the calculation of the scalar THz holography. (a–d) The required phase profiles for generating the cross-pattern holographic image, which are calculated with the pixel size being set to be 25 μm × 25 μm, 50 μm × 50 μm, 100 μm × 100 μm and 200 μm × 200 μm, respectively. (e–h) The calculated holographic images after propagation (distance 50.8 mm). The working frequency in design and calculations is set to be 1.0 THz. P_{unit}, pixel size of the supercells for phase manipulation on the THz hologram.

S3: Measured Scalar THz Holographic Images

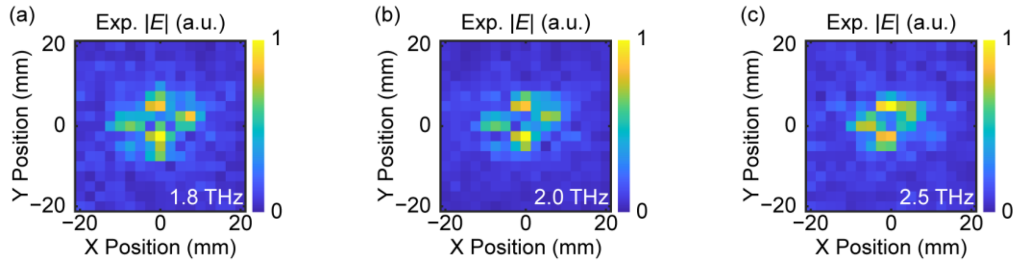

Figure S3. The measured holographic images generated by the metasurface emitter with uniform-type supercells for the scalar THz holography. (a–c) The measured electric fields of the cross-pattern THz holographic images at frequencies of 1.8, 2.0 and 2.5 THz, respectively.

S4: Calculated Scalar THz Holographic Images

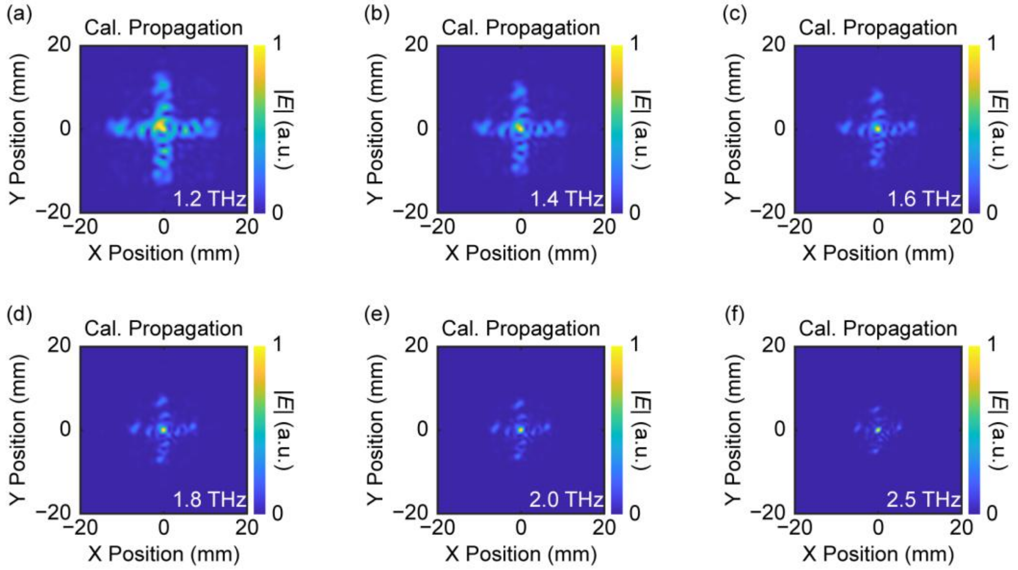

Figure S4. The calculated holographic images of the cross pattern in the scalar design. (a–f) The calculated holographic images after propagation (distance 50.8 mm) at frequencies of 1.2, 1.4, 1.6, 1.8, 2.0 and 2.5 THz, respectively. The working frequency in design is set to be 1.0 THz.

S5: Measured Dual-Polarization THz Holographic Images

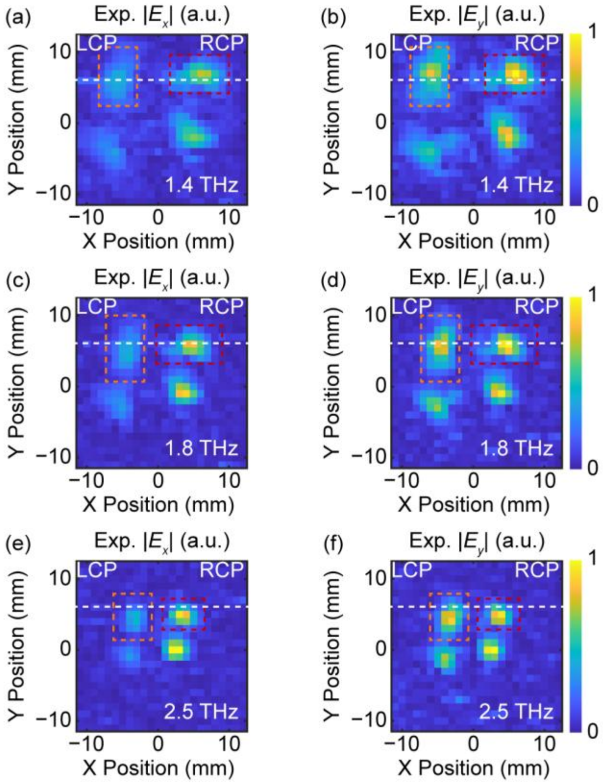

Figure S5. The measured holographic images generated by the metasurface emitter with chessboard-type supercells for the dual-polarization THz holography. (a–f) The measured E_x and E_y electric fields of the dual-polarization THz holographic images at frequencies of 1.4, 1.8 and 2.5 THz, respectively.

S6: Circular Polarization Resolved Dual-Polarization THz Holographic Images

In this section, the circular polarization results of the measured dual-polarization THz holographic image are presented. In the experiments of time-domain THz spectroscopy, the E_x and E_y components of the electric field in the time domain are measured, which is handled by using the fast Fourier transform to obtain the electric fields in the frequency domain. Afterward, the electric field of the left and right circular polarization (LCP and RCP) components can be described by the following equations:

$$E_{\mathrm{LCP}} = \frac{\sqrt{2}}{2}\left(E_x - iE_y\right)\left|\mathrm{L}\right\rangle \quad \text{(S2)}$$

$$E_{\mathrm{RCP}} = \frac{\sqrt{2}}{2}\left(E_x + iE_y\right)\left|\mathrm{R}\right\rangle \quad \text{(S3)}$$

where $\left|\mathrm{L}\right\rangle = \left[1 \quad i\right]^{\mathrm{T}} / \sqrt{2}$ and $\left|\mathrm{R}\right\rangle = \left[1 \quad -i\right]^{\mathrm{T}} / \sqrt{2}$ denote the LCP and RCP vectors, respectively. The LCP- and RCP-resolved electric field magnitude distributions of the dual-polarization THz holographic images at frequency of 2.0 THz are shown in Figure S6a and b. For the LCP-resolved case (Figure S6a), one can observe that the RCP THz component is relatively weak, while the LCP component is obvious. In the RCP-resolved case (Figure S6b), an opposite phenomenon can be observed.

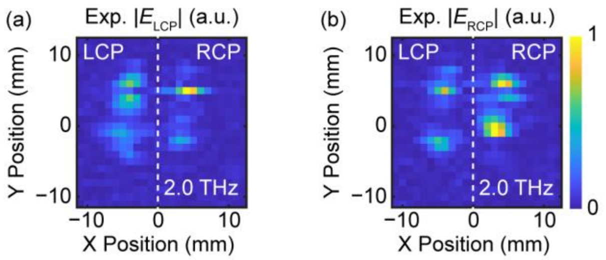

Figure S6. Circular polarization resolved results of the holographic image generated by the metasurface THz emitter with chessboard-type supercells. (a and b) The measured electric fields of the dual-polarization THz holographic images at frequency of 2.0 THz, which are LCP- and RCP-resolved, respectively. LCP/RCP, left or right circular polarization.

S7: Calculated Dual-Polarization THz Holographic Images

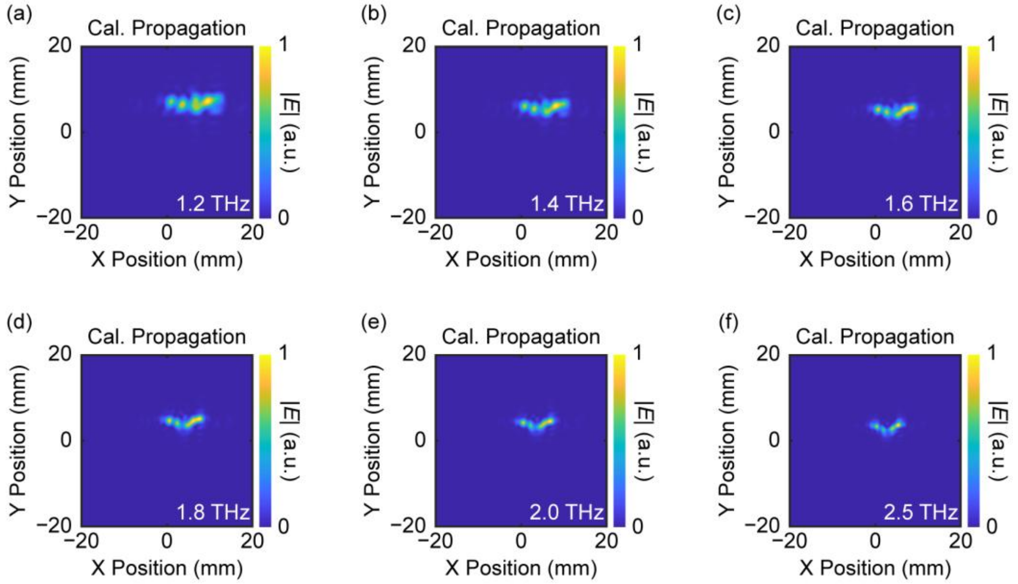

Figure S7. The calculated holographic images of the horizontal bar pattern in the dual-polarization design. (a–f) The calculated holographic images after propagation (distance 50.8 mm) at frequencies of 1.2, 1.4, 1.6, 1.8, 2.0 and 2.5 THz, respectively. The working frequency in design is set to be 1.0 THz.

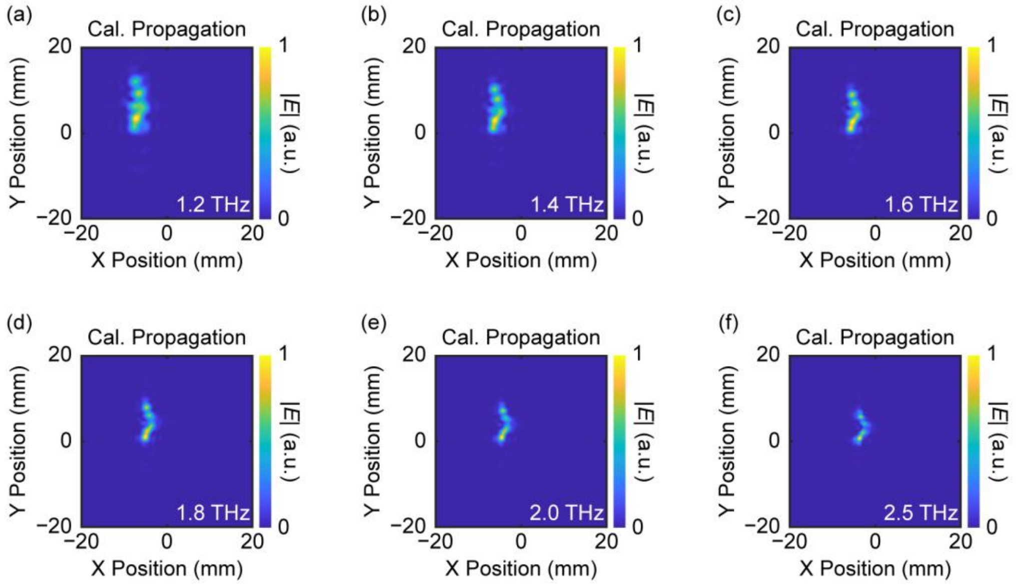

Figure S8. The calculated holographic images of the vertical bar pattern in the dual-polarization design. (a–f) The calculated holographic images after propagation (distance 50.8 mm) at frequencies of 1.2, 1.4, 1.6, 1.8, 2.0 and 2.5 THz, respectively. The working frequency in design is set to be 1.0 THz.

S8: Measured Spectra of THz Waves in Dual-Polarization Holography

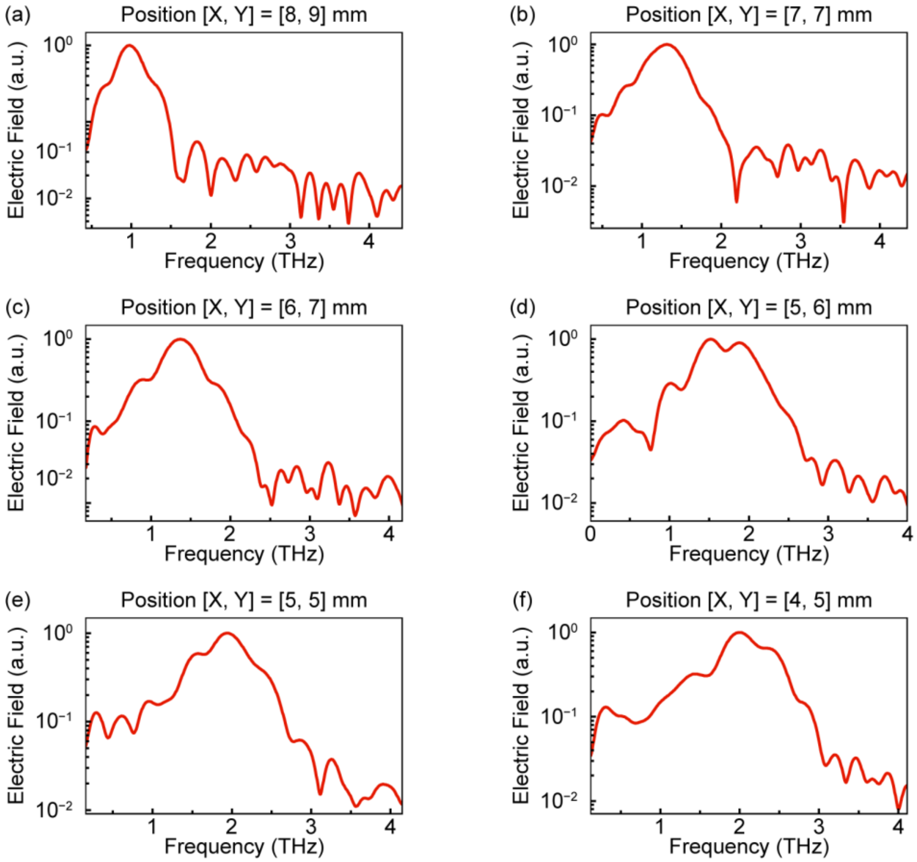

Figure S9. Frequency spectra of the generated THz waves from the metasurface emitter for dual-polarization holography. (a–f) As the sampling position moves from the edge to the center of the holographic image, the central frequency of the spectra gradually increases. It is consistent with the results observed in Figure 5a–h and Figure S5a–f, where the size of intensity profiles in holographic images decreases as the frequency increases.

REFERENCES

1. Goodman, J. W. *Introduction to Fourier optics*, 3rd ed., McGraw-Hill physical and quantum electronics series. Greenwood Village, CO: Roberts and Company publishers, 2005.
